# Supplementary material for: Digital thErapy For Improved tiNnitus carE Study (DEFINE): Protocol for a randomised controlled trial
Source: PLoS One. 2024 Jan 5;19(1):e0292562. doi: 10.1371/journal.pone.0292562 (PMC10769067; doi:10.1371/journal.pone.0292562)
Supplement: S1 File — ‘Full approved protocol for the DEFINE trial dated 10th August 2023’. (DOCX) [file pone.0292562.s002.docx]

**Trial Title: Digital thErapy For Improved tiNnitus carE Study (DEFINE)**

Version 3.0

Date:

Ethics Ref: 23/WM/0146

IRAS Project ID: 328487

Chief Investigator: Dr Matthew Smith (University of Cambridge )

Co Investigators: Dr Jameel Muzaffar (Oto Health)

Dr Oliver Rivero-Arias (Oxford Pharmagenesis/Maths In Health)

Dr Kim Rand (Oxford Pharmagenesis/Maths In Health)

Dr Polly Field (Oxford Pharmagenesis/Maths In Health)

Dr Luke Twelves (Lindus Health)

Sponsor: Oto Health Ltd

4th Floor

Silverstream House

45 Fitzroy Street

London

England

W1T 6EB

Chief Investigator Agreement for the Protocol

I agree to;

● To assume responsibility for the proper conduct of the clinical investigation at this site, and to conduct the trial in compliance with this protocol, any future amendments, and with any other trial’s conduct procedures provided by the sponsor or authorised representatives.

● Not to implement any deviations from or changes to the protocol without agreement from the sponsor and prior review and favourable opinion from the ethics committee and approval from the competent authority, if applicable, except where necessary to eliminate an immediate hazard to the participant(s), or for administrative aspects of the clinical investigation (where permitted by all applicable regulatory requirements).

● To ensure that all persons assisting me with the clinical investigations are adequately informed about the protocol and of their trial-related duties and functions.

Dr Matthew Smith

Chief Investigator

…………………………………………………………………………………………………………

Chief Investigator Signature Date

The signatures below constitute the approval of this protocol and the attachments

Chief Investigator Date

Signature:

Name: Dr Matthew Smith

For and on behalf of the Trial Sponsor: Date

Signature:

Name: Dr Dhiraj Sharma

Position: Oto Founder Associate & NHS Doctor

Statistician: Date

Signature:

Name: Dr. Jan Multmeier

Position: Trial Statistician

**Contents**

[**1 ABBREVIATIONS 6**](#_myvm10jtvsxa)

[**2 STUDY SYNOPSIS 8**](#_b5213363aki9)

[**3 BACKGROUND and RATIONALE 11**](#_kzevfco6ged6)

[**4 OBJECTIVES AND OUTCOME MEASURES 13**](#_c8sq57h8ud2k)

[**5 STUDY DESIGN 14**](#_gac77l4p3cq7)

[**6 PARTICIPANT IDENTIFICATION 14**](#_9rrbdqcn4ymr)

[6.1 Study population 14](#_7wh8sp104z7i)

[6.2 Recruitment of Participants 15](#_yb650ajzire5)

[**7 TRIAL PROCEDURES 16**](#_at7y4e9jjcvl)

[7.1 Digital Applications 16](#_eefw70rpvh0)

[7.2 Pre-screening 16](#_w0iwqc2ly5zi)

[7.3 Informed consent 17](#_39i2dm4ds8g7)

[7.4 Screening and Eligibility Confirmation 17](#_4rkr5rxtgew0)

[7.5 Hearing screening 17](#_41g4soeyks2r)

[7.6 Randomisation 18](#_k2wtrcgoy258)

[7.7 Baseline Measurements (Day 1, up to + 7 days) 19](#_r9maybi6dk4)

[7.8 Administration of intervention 19](#_1ypq4k1b38ik)

[7.9 GP contact and hearing test referral 19](#_szno4e78vlr9)

[7.10 Follow-up (remote) 20](#_no9q3m4f9lq7)

[7.10.1 Month 1 (Study week 4 +/- 7 days) 20](#_41q3or31a18x)

[7.10.2 Month 3 (Study week 12 +/- 14 days) 20](#_7ubsnpfr5ol2)

[7.10.3 Month 6 (Study week 26 +/- 14 days) 20](#_sekuzhyd9226)

[7.10.4 Month 12 (Study week 52 +/- 28 days) 20](#_ssw3h2sek7k5)

[7.11 Early Discontinuation/Withdrawal of Participants 21](#_26eu4omyt1jy)

[7.12 Definition of End of Trial 21](#_u17n5kr3e90g)

[**8. STUDY INTERVENTION 21**](#_qhqap7d0znqa)

[8.1 Intervention Group (intervention description) 21](#_5ut2xsswma9c)

[8.2 Control Group 22](#_9jpp32ujsol0)

[8.3 Concomitant Therapy 22](#_5rgvupdftaaj)

[8.4 Adherence 23](#_sgou3uol841z)

[**9. SAFETY REPORTING 23**](#_pnz1hxg61ek9)

[9.1 Definitions 23](#_1srh4fxepnoy)

[9.2 Causality 24](#_tevt1dn9m5jz)

[9.3 Adverse Event Reporting 24](#_kt23c4q1yzgv)

[9.4 SAE Reporting 24](#_534s8vaa5498)

[9.5 Expectedness 25](#_jqv1w63d70kz)

[9.6 Measures to minimise the occurrence of AEs 25](#_u3rpx5j1op5)

[9.6.1 Tinnitus Distress Monitoring 25](#_c6y9f7ptjq8v)

[**10. STATISTICS 26**](#_yla1z0o7xzie)

[10.1 Description of Statistical Methods 26](#_3f5gtenkicvx)

[10.2 The Number of Participants 27](#_5923fz89lzn3)

[10.3 Criteria for termination of the trial 27](#_43qsv0okab8q)

[10.4 Handling of Missing and Incomplete Data 27](#_xe606fb1slh2)

[**11. HEALTH ECONOMIC EVALUATION 27**](#_uxiuz2jcd6sr)

[11.1 Health Economic Evaluation 27](#_6q8zuxe1e78d)

[**12. PARTICIPANT EXPERIENCE QUALITATIVE ASSESSMENT 29**](#_lqb8nejvrozc)

[12.1 Participant experience 29](#_d5ljx0egt99t)

[12.2 Sampling 29](#_vq01qq2ai5e)

[12.3 Focus group consent 30](#_vl5tnod6bmzo)

[12.4 Focus group design 30](#_x26ch84nfkaz)

[12.5 Focus group topics 30](#_mt5xlh1unjv7)

[12.6 Analysis and outcomes 31](#_y6xscx45d80i)

[**13 DATA MANAGEMENT 31**](#_96hsjn86rm6o)

[13.1 Source Data 31](#_1zl34813lfvi)

[13.2 Access to Data 31](#_sxusedu9bl8j)

[13.3 Data Handling and Record Keeping 31](#_z27gso5xn1ng)

[**14. QUALITY ASSURANCE PROCEDURES 32**](#_ymdhxaw3p7af)

[14.1 Risk Assessment 32](#_kgynkomrugy)

[14.2 Study Monitoring 32](#_eae2snrw4sqq)

[14.3 Study Committees 33](#_gdryhbuqod2v)

[**15. PROTOCOL DEVIATIONS 33**](#_mv84466g3zps)

[**16. SERIOUS BREACHES 33**](#_3dgrukvjhoyh)

[**17. ETHICAL AND REGULATORY CONSIDERATIONS 34**](#_1brovcyk8qlt)

[17.1 Declaration of Helsinki 34](#_l0h2icergq2u)

[17.2 Guidelines for Good Clinical Practice 34](#_tivcwmmbj0du)

[17.3 Approvals 34](#_3izg5ccse8wj)

[17.4 Reporting 34](#_gohvnmseewzs)

[17.5 Participant Confidentiality 34](#_j3do3f3d012t)

[17.6 Expenses and Benefits 35](#_3btacu4n90ag)

[17.7 Patient and Public Involvement and Engagement (PPIE) 35](#_6adiq8r6e9)

[**18. FINANCE AND INSURANCE 35**](#_qxr6ktgtqwyg)

[18.1 Funding 35](#_1vqkj4lsvzt9)

[18.2 Insurance 35](#_s2vsgi4qvv8v)

[18.3 Contractual Arrangements 35](#_bbr0qq91kfpg)

[**19. PUBLICATION POLICY 36**](#_kogqjquf4a5s)

[**20. ARCHIVING 36**](#_fwexurnzejlw)

[**21. REFERENCES 36**](#_z0pe0ch0xa1x)

[**22. APPENDIX 1 – TRIAL FLOW-CHART 39**](#_san4z3k4wgfd)

[**23. APPENDIX 2 - SCHEDULE OF EVENTS TABLE 40**](#_lwlirg5fow56)

[**24. APPENDIX 3 – CONTROL ARM INTERVENTION SPECIFICATION 42**](#_n0nrcmnvmbqc)

[**25. APPENDIX 4 – AMENDMENT HISTORY 44**](#_bqb7ckx8gkfi)

#

#

#

#

#

#

#

#

#

#

#

#

# 1 ABBREVIATIONS

AE Adverse Events

App Application

BTA British Tinnitus Association (now known as Tinnitus UK)

CBT Cognitive Behavioural Therapy

CI Chief Investigator

TSC Trial Steering Committee

e-Consent Electronic consent

eTMF Electronic Trial Master File

EDC Electronic Data Capture

EQ-5D-5L EuroQol Questionnaire

GCP Good Clinical Practice

GDPR General Data Protection Regulation

GP General Practitioner

HRA Health Research Authority

HUI Health Utilities Index

ICF Informed Consent Form

ICH International Conference on Harmonisation

ITT Intention To Treat

NICE National Institute for Health and Care Excellence

PIS Patient Information Sheet

PPS Per Protocol Analysis Set

REC Research Ethics Committee

RCT Randomised Clinical Trial

RN Research Nurse

SAE Serious Adverse Event

SAP Statistical Analysis Plan

SUS System Usability Scale

TFI Tinnitus Functional Index

THI Tinnitus Handicap Inventory

TMG Trial Management Group

TRT Tinnitus Retraining Therapy

UKRI United Kingdom Research and Innovation

KEYWORDS: tinnitus, digital therapeutics, cognitive behavioural therapy (CBT), health economics.

# 2 STUDY SYNOPSIS

| Title | Digital thErapy For Improved tiNnitus carE |
| --- | --- |
| Short title | DEFINE |
| Chief Investigator | Dr Matthew Smith |
| Background | Tinnitus is a common condition, affecting approximately 15% of UK adults. It is usually perceived as a buzzing or ringing in the ears, without a stimulus in the outside world. There is currently no cure, but cognitive behavioural therapy (CBT) has been shown to be effective in reducing self reported distress and illness severity. Unfortunately, access to CBT is limited, with significant healthcare costs associated with provision and additional costs to people with tinnitus including time away from work, travel and lost productivity. |
| Primary objective | To assess whether Oto’s digital tinnitus programme is no worse at reducing self-reported tinnitus severity as therapist-delivered CBT |
| Secondary objectives | - Assess whether Oto’s digital tinnitus programme is superior to therapist-delivered CBT in reducing self-reported tinnitus severity - Assess the self-reported impact of Oto’s digital tinnitus programme on aspects of participants tinnitus experience - Assess the impact on overall health-related quality of life of Oto’s digital programme and human delivered CBT - Assess the usability of the Oto digital programme - Explore participants views of the impact and usability of Oto’s digital programme - Understand the health economic consequences of using a digital therapeutic for tinnitus - Assess the number of adverse events between the groups - Assess intervention Adherence |
| Study design | An open-label, non-inferiority, prospective, parallel design, randomised-controlled study assessing the Oto smartphone tinnitus management programme versus therapist delivered CBT in achieving improvement in self-reported tinnitus severity in adults. |
| Phase | III |
| Setting | Fully decentralised study with participants recruited remotely. Interventions and assessments delivered remotely via the Oto health smartphone app, video calls and the Lindus Health platform. |
| Recruitment Strategy | Patients will be recruited centrally by the Lindus Health research team, with Lindus Health acting as a research site. Patients will be identified via primary care, advertising on social media and to patient communities, in addition to healthcare professionals directing potential participants to the trial website. Patients can receive £40 for taking part in the trial. |
| Primary Outcome | Total TFI score (Score of 0-250 with high score indicating worse outcome) 6 months from starting therapy |
| Secondary Outcomes | 1) Total TFI and TFI subscale scores at 1, 3 and 12 months  2) Participant health-related quality of life measured using the EuroQol EQ-5D-5L and the Health Utilities Index Mark 3 (HUI3) responses and summary scores at 3, 6 and 12 months follow-up from starting therapy  3) Adverse events from randomisation to 3 months  4) System Usability Scale scores at 3 months  5) Qualitative feedback from focus groups and semi structured interviews at 3, 6 and 12 months  6) Cost-utility analysis synthesising costs and QALYs over trial period  7) Assess intervention adherence at 3 and 6 months |
| Number of participants | 196 participants, randomised in a 1:1 ratio |
| Eligibility criteria | Inclusion Criteria   1. Aged 18 years or over 2. Experienced tinnitus symptoms for at least 3 months and self-assessed as having a significant impact on quality of life. 3. Have access to a smartphone 4. Able and willing to give consent for the study prior to participation 5. Able to speak and read English to a sufficient level.   Exclusion Criteria   1. Tinnitus with potential “red flag” symptoms i.e. unilateral or pulsatile tinnitus (assessed via a red flag checklist during the screening call) 2. Significant mental health problems e.g. history of suicidal ideation or requirement for psychiatric/psychological support beyond Primary Care level 3. Have required hospitalisation for depression or taking antipsychotic drug 4. Currently taking part in another clinical trial for hearing/tinnitus 5. Awaiting surgical intervention for hearing/tinnitus 6. Previously undergone tinnitus therapy of any type e.g. CBT or TRT 7. Pregnant and breastfeeding women   Additional Note: Patients with hearing aids are eligible to participate in the study |
| Intervention | The Oto digital programme for tinnitus. This involves direct access to a digital multi-modal therapy package incorporating CBT, physical therapy, mindfulness and relaxation therapy. |
| Comparator | Current standard of care (1-1 therapist-delivered CBT). |
| Duration of study | 18 months |
| Randomisation / Blinding | Randomised 1:1 by computer algorithm and stratified by age, sex and level of hearing loss. Unblinded. |
| Statistical analysis | Non-inferiority |
| Intervention Adherence | Number of participants in each group completing therapy programme by 3 months |

# 3 BACKGROUND and RATIONALE

3.1 Background

Tinnitus is the perception of sound in the absence of an external stimulus. Tinnitus is very common with approximately 15.3% of the UK population experiencing prolonged tinnitus with 5.4% considering this bothersome^1^. Prevalence varies with age, with approximately 10% of younger adults, 14% of middle aged adults and 24% of older adults experiencing tinnitus^2^.

Treatment options for people with tinnitus are currently suboptimal. The approach with the strongest evidence base is Cognitive Behavioural Therapy (CBT), with guided self-directed CBT showing the largest effect size and having the highest likelihood of being ranked first in improving tinnitus health-related quality of life (75%), depression (83%), and anxiety (87%)^3^. Current NHS provision as delivered by Ear, Nose and Throat Surgeons, Audiologist and Hearing Therapists, focuses on ensuring there are no concerning features, such may indicate cerebellopontine angle pathology or systemic health problems. Once these are ruled out, patients may be offered referral for tinnitus therapy. Provision of this therapy, both in terms of access and method of delivery, is inconsistent. Current NICE guidance advocates a combination of reassurance, mitigation of any underlying cause if possible, optional hearing aiding if concomitant hearing loss is present, self-care advice and consideration of referral for psychological therapy^4^.

Current NHS tinnitus therapy provision is typically based on the principles outlined in Tinnitus Retraining Therapy (TRT), though some elements of Cognitive Behavioural Therapy (CBT) and relaxation may also be incorporated. Provision outside the NHS is more varied, usually featuring longer term support and contact and a wider range of therapeutic techniques. This provision is typically on a one-to-one basis and lasts between one and ten sessions. However, many people with tinnitus do not access such support and an opportunity exists to expand provision to this underserved group as well as supplementing and optimising current provision for those able to access therapeutic support.

The proliferation of smartphone technology has led to the development of a number of app-delivered therapies for a variety of health conditions, partially stimulated by the COVID-19 pandemic^5^. These have been deployed most extensively for mental health indications with both primary research and evidence synthesis to support their use^6^. At present a number of such apps are available for tinnitus, though they predominantly rely on sound therapy (masking)^7,19^.

Oto is a novel multimodal smartphone app-delivered approach to tinnitus. It combines patient education, CBT, relaxation, mindfulness and sound therapy in a customisable package. The self-paced and self-administered delivery method also allows patients to avoid taking time off work and eliminates travel time to a hospital. In contrast to traditional therapy, the app removes limits on the number of individuals that can be served at the same time, and has the potential to significantly advance capacity at a time when waiting lists are under severe pressure.

3.2 Rationale

Tinnitus presents a large burden on society: with 1m+ GP appointments for tinnitus, the NHS spends c.£750m annually, and with loss of productivity among other costs, tinnitus is responsible for an annual loss to the economy of £2.7b in the UK^16^.

The main aim of this study is to assess whether Oto’s digital therapeutic is no less effective than current standard of care (human delivered CBT) as defined by change in self-reported tinnitus severity, as measured by the Tinnitus Functional Index at six months from starting therapy. A cost-utility analysis will be undertaken to evaluate the potential of the Oto app to represent value for money for the NHS and society more widely.

# 4 OBJECTIVES AND OUTCOME MEASURES

4.1 Randomised Controlled Trial

Primary Objective

| **Objective** | **Outcome Measure** | **Timepoint(s) of evaluation** |
| --- | --- | --- |
| Assess whether Oto’s digital tinnitus programme is as effective at reducing self-reported tinnitus severity as therapist- delivered CBT | Total TFI score | Primary outcome is total TFI score at 6 months  TFI evaluated at:  Baseline, 1, 3, 6 and 12 months |

Secondary Objectives

| Objective | Outcome Measure | Timepoint(s) of evaluation |
| --- | --- | --- |
| Assess whether Oto’s digital tinnitus programme is superior to therapist-delivered CBT in reducing self-reported tinnitus severity | Total TFI score | Total TFI score at 6 months |
| Assess the self-reported impact of Oto’s digital tinnitus programme on aspects of participants tinnitus experience | 8 Tinnitus Functional Index subscales (Intrusive, Sense of Control, Cognitive, Sleep, Auditory, Relaxation, Quality of Life and Emotional). | Baseline, 1, 3, 6 and 12 months |
| Assess the impact on overall health-related quality of life of Oto’s digital programme and therapist delivered tinnitus therapy | EuroQol EQ-5D-5L and Health Utilities Index Mark 3 (HUI3) | Baseline, 3, 6 and 12 months |
| Assess the usability of the Oto digital programme | System Usability Scale | 3 months |
| Explore participants views of the impact and usability of the two interventions | Thematic analysis of focus groups and semi structured interviews | 3, 6 and 12 months |
| Understand the health economics of the two for tinnitus | Cost-utility analysis | Over trial period covering from baseline to 12 months |
| Assess the number of adverse events between the groups | eCRF | Randomisation to 3 months |
| Assess intervention Adherence | Number of participants completing the intervention | Number of participants starting the intervention at baseline compared to those completing the intervention at 3 and 6 months |

# 5 STUDY DESIGN

5.1 Study Type

Prospective, open-label, randomised controlled, parallel design with an active control.

5.2 Duration

Participants will be assigned to the intervention or standard usual care for 12 months, with an additional 1-week onboarding prior to this.

# 6 PARTICIPANT IDENTIFICATION

## 6.1 Study population

Adults with tinnitus that has persisted for at least 3 months and is self-assessed as impacting quality of life. Participants must not have previously undergone tinnitus therapy (TRT or CBT by any delivery method).

Participants may be identified by:

- searches of primary care electronic health records
- potential participants may be directed to the study website by any clinician seeing them for the condition
- participants can self present using the website in response to social media or patient community advertising.

6.1.1 Inclusion Criteria

Participants must meet all the following criteria:

Inclusion Criteria

1. Aged 18 years or over
2. Experienced Tinnitus symptoms for >= 3 months and self-assessed as impacting quality of life
3. Have access to a smartphone
4. Able and willing to give consent for the study prior to participation
5. Able to speak and read English to a sufficient level

6.1.2 Exclusion criteria

Participants meeting any of the following criteria are not eligible for inclusion in the study:

1. Tinnitus with potential “red flag” symptoms (e.g., unilateral or pulsatile tinnitus)
2. Significant mental health problems e.g. history of suicidal ideation or requirement for psychiatric/psychological support beyond Primary Care level
3. Have required hospitalisation for depression or taking antipsychotic drug
4. Currently taking part in another clinical trial for hearing/tinnitus
5. Awaiting surgical intervention for hearing/tinnitus
6. Previously undergone tinnitus therapy of any type e.g. CBT or TRT
7. Pregnant or breastfeeding women

The rationale for excluding “Pregnant and breastfeeding women” is that this population was not able to be included in the trial insurance coverage.

## 6.2 Recruitment of Participants

Potential participants will be identified via several different mechanisms:

1. Clinicians working at GP practices (Participant Identification Centres - PICs) within the Lindus Health Primary Care Network (this covers GP groups, networks or single practices across the UK) will be informed of the study and will describe the study to patients presenting tinnitus symptoms of at least 3 months. If the patient is interested in knowing more about the study they will be directed to the trial website where more information about the trial will be displayed and have the choice to sign up to receive the patient information sheet and a video explaining the trial.
2. The electronic medical records of patients at participating primary care practices (Participant Identification Centres - PICs) within the Lindus Health Primary Care Network. GP practices will be provided with a search for their registers to identify potential participants, who will be invited to take part in the trial via a text message directing them to the trial webpage where they will be able to receive more information about the trial and have the choice to sign up to receive the patient information sheet and a video explaining the trial.
3. Social media advertising on Facebook and other platforms will be used to highlight the trial to individuals with tinnitus. Adverts will make clear that the study does not replace the need to seek medical advice. Adverts will direct potential participants to the study website, where more information about the trial will be displayed and have the choice to sign up to receive the patient information sheet and a video explaining the trial.

All participants will receive up to £40 for taking part in the trial.

# 7 TRIAL PROCEDURES

Trial procedures are detailed in the Trial Flow Chart (Appendix 1). The study will be conducted in keeping with the Good Clinical Practice guidelines and regulations.

## 7.1 Digital Applications

For the duration of the trial participants will be asked to record their responses to questionnaires directly into the Lindus Health Electronic Data Capture (EDC) platform, via a participant survey link, which will be emailed and/or texted to them. The participants in the smartphone delivered CBT arm will also use the Oto tinnitus app. Certain fields may require duplicate data entry between the two digital systems, but it is essential that data on the Lindus Health platform is completed fully.

The Lindus Health team will have access to the EDC data and will review this on a daily basis (excluding weekends). Missing data will trigger an alert message to the participant via text/email. If no response is entered, a daily reminder will be triggered for 3 days, after which point the Lindus Health team will contact the participant to assess if they wish to withdraw from the study. If a participant wishes to withdraw then they will be advised to arrange an appointment with their GP or healthcare provider to discuss other treatment options (i.e. return to standard care), and a reason for withdrawal will be documented. Free access to the app will continue for participants in the Oto arm, or be offered free for 12 months from completion of the study for participants in the control group.

## 7.2 Pre-screening

The potential participant will be directed to the trial web page, where they will be informed about the trial via the study website. If interested in taking part, they will complete an online pre-screening form to assess their eligibility for the trial. If potentially eligible and once they submit the form, the Patient Information Sheet (PIS) and Informed Consent Form (ICF) will be emailed directly to the participant, as well as a video explaining the trial, and they will be asked to book a telephone/video call with a member of the central trial team.

## 7.3 Informed consent

Prior to consent, the patient will have received via email the PIS, ICF and video describing the trial. They will have had the opportunity to read through the details relating to the exact nature of the trial, the known side-effects and any risks involved in taking part. It will be clearly stated that the participant is free to withdraw from the study at any time for any reason without prejudice to future care, without affecting their legal rights, and with no obligation to give the reason for withdrawal. It will be confirmed that potential participants have reviewed the PIS.

Participants will be asked to provide informed consent only after a discussion with a suitably qualified and delegated member of the central trial team and prior to randomisation. Adequate time will be given to the participant to consider the information and to ask any questions about the trial before deciding whether to participate. If the participant is willing, informed consent will be obtained. The participant will provide their electronic signature at the end of the e-consent form and the central trial team member will countersign the form. A copy of the fully executed ICF will be sent to the participant by email and an electronic copy retained by Lindus Health in a secure area with restricted access. The participant’s GP will be informed of their involvement in the trial.

Identifiable participant details (confidential details, including name and date of birth) will be held in a database separated from the research database after receiving participants consent. The research database will never hold personal identifiable information. Automatic reminders will be sent to participants by doing a one-time recall of the identifiable information by matching the unique study ID.

## 7.4 Screening and Eligibility Confirmation

Screening will be conducted remotely by a member of the central trial team, during the same telephone/video call where informed consent is obtained. A member of the central trial team will use the medical history obtained from the participant to confirm that the participant meets the inclusion and exclusion criteria. A record of screen failures that do not meet the inclusion/exclusion criteria will be retained.

Given the low risk of intervention, participant eligibility will be assessed by a member of the central trial team who will assess this against the self-reported medical criteria to determine suitability for the study.

## 7.5 Hearing screening

When the consent form is sent out participants will be asked in advance to have headphones available at the time of their screening and consent phone call. For participants who consent to trial involvement they will be directed to download a smartphone application to screen their hearing for the sole purpose of stratification at randomisation. Participants will be asked if they have an ios or Android-based smartphone. Those with an ios-based device will be asked to download the ‘Easy Hearing Test’ application (produced by Hiroaki Ito) . Those with an Android-based phone will be asked to download the ‘Hearing test’ app (produced by e-audiologica).

Both applications are free to the user and do not require the user to register or share personal identifiable data.

Participants will be given instructions on use of the apps, and allowed to complete the hearing test for both ears, which typically takes around 5 minutes. The participant will then share the hearing test audiogram result with the member of the trial team leading the video call, this image will also be screen captured and stored in the trial database. The trial team will store the data associated with the participants trial ID and record the mean pure tone threshold at 0.5, 1, 2 and 4kHz. All participants will be encouraged to seek formal audiometric testing (see below) rather than to rely on the screening hearing test as it will be made clear that these results are not diagnostic. We have validated these applications ourselves in the home environment, and demonstrated good correlation with hospital pure tone audiogram results (unpublished data).

## 7.6 Randomisation

Following confirmation of eligibility, the participant will be randomised using a secure, fully validated and compliant web-based randomisation system called Sealed Envelope.

Participants will be randomised into one of the two groups at a ratio of 1:1, stratified by

1. Age (<40, >=41-<=60, >61)
2. Sex (Male, Female)
3. Pure tone average threshold based on home hearing test (mean threshold at 0.5, 1, 2, 4 kHz (‘None’ <=15dB, ‘Slight’ 16-25dB, ‘Mild’ 26-40dB, ‘Moderate’ 41-55dB, ‘Moderately severe’ 56-70dB, ‘Severe’ 71-90dB, ‘Profond‘ 91dB+)

1. Control arm: participants will receive one to one therapist-delivered tinnitus therapy from an audiologist/hearing therapist. The specification for the tinnitus therapy to be provided as a control intervention can be found in Appendix 3. This will be delivered remotely, and will consist of a minimum of 1 session, with further sessions at the discretion of the participant and therapist up to a maximum of 6. Therapy will begin within +/- 1 week of randomisation. There will be a panel of therapists selected to deliver the therapy, with all individuals having experience of NHS tinnitus care. The therapy provided will broadly conform to the standard NHS treatment as outlined below.
2. Intervention arm: participants will receive the Oto tinnitus programme (smartphone app delivered CBT). This includes an evaluation of symptoms and tinnitus severity, CBT, mindfulness and other interventions. More details on the Oto app can be found here: <https://www.joinoto.com>

A member of the central trial team will inform the participant during their eligibility call or directly call the participant to inform them of their randomisation allocation, and they will also be sent an email confirmation.

Randomisation represents trial day 1 and outcome assessments will be timed from this point.

## 7.7 Baseline Measurements (Day 1, up to + 7 days)

Once randomisation has been confirmed and prior to starting the intervention, participants will report baseline measurements in their survey which will include but not limited to:

- Complete information on previous treatment for tinnitus and costs already incurred e.g. over the counter supplements, hearing aids
- Complete TFI questionnaire
- Complete EQ-5D-5L and HUI3 questionnaires

## 7.8 Administration of intervention

Following baseline data collection the participant will be granted access to either the trial intervention or control intervention. Access to the Oto app will be immediate. The first face-to-face CBT appointment will be scheduled within +/- 1 week.

## 7.9 GP contact and hearing test referral

With the participant’s permission provided on the trial consent form, a letter will be sent to the participant’s GP to inform them that the individual has entered the DEFINE trial.

It will be recommended to all participants both on the screening phone call and in the following email that they speak to their GP and request referral for a formal pure tone audiogram hearing test. The method of referral will be at the discretion of the GP, but it will be recommended that this is achieved via referral to an approved high street provider under the Any Qualified Provider (AQP) scheme.

## 7.10 Follow-up (remote)

### 7.10.1 Month 1 (Study week 4 +/- 7 days)

To collect data to include but not limited to:

- Complete TFI questionnaire
- Adverse events
- Any change in medication (particularly medications known to be associated with tinnitus, e.g. Aspirin, diuretics) or introduction of hearing aid

### 7.10.2 Month 3 (Study week 12 +/- 14 days)

To collect data to include but not limited to:

- Complete TFI questionnaire
- Complete EQ-5D-5L and HUI3 questionnaires
- Adverse events
- Complete System Usability Score (SUS)
- Complete questionnaire on primary and community healthcare appointments and A&E visits related to tinnitus since week 4
- Complete questionnaire on personal out-of-pocket tinnitus related costs, any time-off work and travel information to appointments incurred since week 4
- Any change in medication (particularly medications known to be associated with tinnitus, e.g. Aspirin, diuretics) or introduction of hearing aid

### 7.10.3 Month 6 (Study week 26 +/- 14 days)

To collect data to include but not limited to:

- Complete TFI questionnaire
- Complete EQ-5D-5L and HUI3 questionnaires
- Complete questionnaire on primary and community healthcare appointments and A&E visits related to tinnitus since week 12
- Complete questionnaire on personal out-of-pocket tinnitus related costs, any time-off work and travel information to appointments incurred since week 12
- Any change in medication (particularly medications known to be associated with tinnitus, e.g. Aspirin, diuretics) or introduction of hearing aid

### 7.10.4 Month 12 (Study week 52 +/- 28 days)

To collect data to include but not limited to:

- Complete TFI questionnaire
- Complete EQ-5D-5L and HUI3 questionnaires
- Complete questionnaire on primary and community healthcare appointments and A&E visits related to tinnitus since week 26
- Complete questionnaire on personal out-of-pocket tinnitus related costs, any time-off work and travel information to appointments incurred since week 26
- Any change in medication (particularly medications known to be associated with tinnitus, e.g. Aspirin, diuretics) or introduction of hearing aid

## 7.11 Early Discontinuation/Withdrawal of Participants

Each participant has the right to withdraw from the study at any time. In addition, the participant’s GP or trial Sponsor may discontinue the participation of a participant from the study at any time, if this is considered necessary for any reason including:

● Pregnancy

● Ineligibility

- Participant decision

● Inability to comply with study procedures

● Withdrawal of consent

Following participant withdrawal, no further clinical assessments will be performed. The participant will be contacted by a member of the central team and a reason for withdrawal will be requested and documented. All data recorded prior to withdrawal will be analysed in the intention-to-treat population.

## 7.12 Definition of End of Trial

The end of the study is defined as the last participant’s last data capture.

# 8. STUDY INTERVENTION

## 8.1 Intervention Group (intervention description)

The Oto Tinnitus Programme is a digital therapeutic combining CBT with mindfulness, patient education and physical therapy (physical stretches/exercises). Oto’s programme offers a series of daily therapy sessions, where the user listens to recorded audio content that takes them through education, cognitive exercise or a tinnitus-specific meditation, and optional 1-1 chat support. Users work their way through progressive modules using a spiral curriculum, where the sessions build on techniques and exercises learned in previous sessions. The programme lasts approximately 6 weeks, using the app 4-5 times per week, which evidence suggests is sufficient time for a therapeutic difference. Users can personalise their therapy by listening to additional modules and sounds from the sound library.

Participants are expected to have completed the intervention in approximately six weeks.

## 8.2 Control Group

Those allocated to the control arm of the study will undertake one to one tinnitus tinnitus therapy from a trained Audiologist/Hearing Therapist/Psychologist via video calls. This is not a direct mirror of NHS practice as patients will have rapid access to therapy, rather than typical long waiting times from GP referral. Participants in the control group will agree to not use Oto or an alternative tinnitus app for the duration of the study.

This control intervention will consist of a minimum of 1 tinnitus therapy session, with further sessions at the discretion of the participant and therapist up to a maximum of 6. Therapy will begin within 1 week of randomisation. There will be a panel of therapists selected to deliver the therapy. The therapy will include several elements of current standard care formulated into a personal management plan. :

- Assessment of participant needs
- Participant education
- CBT
- Relaxation therapy
- Signposting to hearing aid fitting and other support

In order to facilitate retention, participants in the control group will be offered the Oto Tinnitus Programme at the end of the 12-month RCT period.

Therapists will provide data to Lindus Health on attendance and number of sessions for each participant.

## 8.3 Concomitant Therapy

Participants will be directed to contact their GP for hearing aid referral to a rapid access service such as is provided by accredited ‘Any Qualified Provider’ (AQP) high street audiologists. Any form of hearing aid provision including tinnitus maskers will be permitted and this will be recorded via participant questionnaire.

No medication is administered within the study, nor are any medications prohibited.

## 8.4 Adherence

The number of participants continuing with the intervention at 3 and 6 months will be assessed, as a measure of adherence to the Oto Programme.

# 9. SAFETY REPORTING

## 9.1 Definitions

| Adverse Event (AE) | Any untoward medical occurrence in a participant to whom a medicinal product has been administered, including occurrences which are not necessarily caused by or related to that product. |
| --- | --- |
| Serious Adverse Event (SAE) | A serious adverse event is any untoward medical occurrence that:   - results in death - is life-threatening - requires inpatient hospitalisation or prolongation of existing hospitalisation - results in persistent or significant disability/incapacity - consists of a congenital anomaly or birth defect*.   Other ‘important medical events’ may also be considered a serious adverse event when, based upon appropriate medical judgement, the event may jeopardise the participant and may require medical or surgical intervention to prevent one of the outcomes listed above.  NOTE: The term "life-threatening" in the definition of "serious" refers to an event in which the participant was at risk of death at the time of the event; it does not refer to an event which hypothetically might have caused death if it were more severe.  *NOTE: Pregnancy is not, in itself an SAE. In the event that a participant or his/her partner becomes pregnant whilst taking part in a clinical trial or during a stage where the foetus could have been exposed to the medicinal product (in the case of the active substance or one of its metabolites having a long half-life) the pregnancy should be followed up by the investigator until delivery for congenital abnormality or birth defect, at which point it would fall within the definition of “serious”. |

NB: to avoid confusion or misunderstanding of the difference between the terms “serious” and “severe”, the following note of clarification is provided: “Severe” is often used to describe intensity of a specific event, which may be of relatively minor medical significance. “Seriousness” is the regulatory definition supplied above.

## 9.2 Causality

The relationship of each adverse event to the intervention must be determined by a medically qualified individual according to the following definitions:

- **Unrelated** – where an event is not considered to be related to the intervention
- **Possibly** – although a relationship to the intervention cannot be completely ruled out, the nature of the event, the underlying disease, concomitant medication or temporal relationship make other explanations possible
- **Probably** – the temporal relationship and absence of a more likely explanation suggest the event could be related to the intervention.
- **Definitely** – the known effects of the intervention, its therapeutics class or based on challenge testing suggest that the intervention is the most likely cause.

All AEs (SAEs) labelled possibly, probably or definitely will be considered as related to the intervention.

## 9.3 Adverse Event Reporting

We do not anticipate that the study intervention (Oto Digital Tinnitus Programme) should result in any adverse events (AEs) but include this section in case such events are reported so that they can be considered for causal links to the study. Only AEs that are clinically judged by the research site PI (a medical Doctor at Lindus Health) as being caused by the trial intervention will be reported to the REC that gave favourable opinion. Participants will be asked in their follow up if they have experienced any side effects or medical events while participating in the DEFINE trial. Participants may also report potential (S)AEs via telephone calls or email correspondence with the central trial team. AEs will be collected from randomisation until and including the 3 month follow-up contact.

## 9.4 SAE Reporting

Any AEs which fall under the definition of a SAE will be reported to the Chief Investigator or designated person within 24 hours, to be reviewed for relatedness and expectedness. However, hospitalisations for elective treatment of a pre-existing condition do not need reporting as SAEs.

Reports of related and unexpected SAEs (in the opinion of the Chief Investigator or designated person) will be reported to the REC within 15 days of the CI and/or central trial team becoming aware of the event. The Chief Investigator will also notify the Sponsor of all study SAEs.

Any pregnancy occurring during the study will result in the immediate discontinuation of the participant and the Sponsor should be notified within 24 hours so appropriate follow-up is conducted. Pregnancy itself is not an AE.

All SAEs will be recorded in the Citrus platform and any concerns raised immediately with the CI and may be tabled for discussion at the regular Trial Management Group meeting or referred to an independent Trial Steering Committee (TSC) for review.

## 9.5 Expectedness

We do not anticipate any serious adverse events due to the digital intervention, data collection or the focus groups..

The adverse events described below are expected to occur in this participant population and will not be classified or reported as SAEs unless felt to be directly related to the study intervention or qualitative work. This will be judged by the CI and ratified by the TSC.

- Fluctuation in self reports of severity of tinnitus and mood relating to this.

There are no known side effects relating to CBT, but as tinnitus can be a psychologically distressing condition patients will be made aware that they need to seek medical attention from the GP or other services available to them (such as 111, Accident & Emergency etc) in the event that their psychological state deteriorates, e.g., they develop suicidal ideation.

## 9.6 Measures to minimise the occurrence of AEs

### 9.6.1 Tinnitus Distress Monitoring

Participants will self-monitor their tinnitus related distress via the TFI at follow up intervals. Throughout the trial they will be asked to use the traffic light system (see Appendix 4) to ensure that they seek medical care as required. To further prevent any potential safety events, the central Lindus Health team will also monitor TFI scores at follow up visits and using the traffic light system (see Appendix 3), alert the participant and their GP to any TFI measurements which require follow-up (this is defined as any TFI score of 180 or above). All participants will also receive a phone call from a member of the clinical central trial team, 4 weeks after randomisation, to answer any queries and to specifically identify any potential issues relating to psychological distress.

# 10. STATISTICS

The Statistician will be responsible for the statistical analysis, in accordance with the Statistical Analysis Plan. The SAP will be prepared separately, but an overview of the statistical methodology is provided here.

## 10.1 Description of Statistical Methods

The primary analysis will be conducted on the intention-to-treat (ITT) population. In addition to this, a per-protocol analysis will also be planned as a sensitivity analysis. Compliance will be defined as a minimum of 60 minutes of total use for the intervention group, and one or more sessions for the CBT group. Participants reporting poor compliance will be recorded and these participants will be included in the ITT analysis. Adherence to the smartphone app delivered programme will be monitored through the app with results analysed by intention-to-treat (ITT).

The primary outcome will be reduction in total TFI score at the 6 month point in the ITT population. Non-inferiority will be assessed by constructing a 95% confidence interval around the mean difference in TFI reduction between the groups, and comparing the -upper bound of the confidence interval to the pre-specified non-inferiority margin.

A secondary analysis of the primary outcome will examine significant differences between the control and intervention group’s TFI score at 6 months using a general linear model or a comparable suitable method, using the stratification variables and other potential confounding variables as covariates.

As a sensitivity analysis, the primary outcome will be analysed using the Per-Protocol (PP) population, which consists of participants complying with the treatment regimen. Compliance will be defined as a minimum of 60 minutes of total use for the intervention group, and one or more sessions of CBT for the control group.

Secondary outcomes will be compared between groups and over time with mixed-effects regression models for repeated measures, using the stratification variables and other potential confounding variables as covariates. Logistic models will be used for binary outcomes, and Gaussian models for continuous outcomes. An interim analysis may be conducted once a sufficient number of participants have completed the 6 month milestone. The results of the interim analysis do not influence the continuation of the trial.

## 10.2 The Number of Participants

The present study aims to show that smartphone app-delivered tinnitus therapy is not inferior to CBT in reducing tinnitus symptoms, assessed by the TFI score, after 6 months.

The required sample size was calculated based on a non-inferiority trial design with a non-inferiority margin of 13 points on the TFI scale, as used by two previous studies 9 10, and a standard deviation of 31 points. Assuming a power (1-β) of 90% and a significance level (α) of 2.5%, the sample size was determined as 90 participants per group. To account for a potential attrition rate of 10%, we increased the total sample size to 196 participants.

## 10.3 Criteria for termination of the trial

It is not anticipated that the trial will be suspended or terminated unless in the event of safety concerns or futility such as poor recruitment rates. The study may resume once concerns are addressed and with agreement from the CI, Sponsor, TSCand Ethics committee.

## 10.4 Handling of Missing and Incomplete Data

The Citrus EDC provides real-time notifications to the trial team to inform them of any missing data to minimise the risk of missing data. For patient reported outcomes, the EDC will notify the study team if assessments are overdue so the study team can follow up and collect these outcomes (the trial team will contact the patient up to 3 times). If a patient reported outcome is not completed within the scheduled window, the data will be considered missing with reasons provided. For clinician reported outcomes the EDC system will flag if an outcome is missing. Missing data will be managed according to Lindus Health SOPs. Missing data will be reported with reasons given where available and imputed using multiple imputation for the ITT population. Depending on the extent of missing values, further investigation may be made into the sensitivity of the analysis results to the method(s) specified.

# 11. HEALTH ECONOMIC EVALUATION

## 11.1 Health Economic Evaluation

A health economic evaluation forms an integral part of the trial and will be conducted from the perspectives of the NHS, personal and the society. The economic evaluation will take the form of a cost-utility analysis with a one-year time horizon using quality-adjusted life years (QALYs) as the main outcome measure. Although the clinical study has a non-inferiority design and our hypothesis is that QALYs will be similar between the Oto app and standard care, there is no prior hypothesis that costs will be similar between groups. Therefore, a standard economic evaluation framework will be used to synthesise differences in costs and QALYs between study arms using the incremental cost-effectiveness ratio (ICER)^8^.

We will follow all participants from baseline to 12 months and will collect information about the delivery of the intervention (app and standard care), primary and community health care, visits to accident and emergency (A&E) and prescribed medications. We will focus on the collection of resource utilisation related to tinnitus. Data will be collected using bespoke questionnaires that will be embedded into the Lindus Health EDC platform for participant completion at regular intervals during follow-up (weeks 12, 26 and 52). Unit costs from recognised national sources including the new NHS National Cost Collection, the Personal and Social Services Research Unit (PSSRU), and the NHS Electronic Drug Tariff will be used to value NHS health care resource use. To understand the burden beyond the health service, we will ask participants about any related out-of-pocket expenses (e.g. hearing aids, complementary and alternative paid treatments) and any time away from paid employment due to the condition.

Tinnitus is known to impair aspects of daily health-related quality of life (HRQoL) and such information will be collected in the study. For the purpose of the economic evaluation, HRQoL instruments that are preference-based are required for QALY estimation. In this study we will administer the EuroQol EQ-5D-5L and the Health Utilities Index Mark 3 (HUI3)^9^. Both questionnaires are short and the EQ-5D-5L covers descriptive levels for five dimensions (mobility, self-care, usual activities, pain/discomfort, and anxiety/depression) whereas the HUI3 covers descriptive levels for eight dimensions (vision, hearing, speech, ambulation, dexterity, emotion, cognition and pain). The health state described in each measure is converted into a single value on a scale where death is anchored at 0 and perfect health at 1 using value sets. The EuroQol EQ-5D is currently the preferred preference-based instrument to measure HRQoL in adults by the National Institute for Health and Care Excellence (NICE) and we will be using the interim 5L- > 3L mapping tool to estimate utility values^10^. To calculate utility values from HUI3 responses we will use the Canadian tariff as a UK-specific value set is currently not available. The questionnaires will be administered at baseline and at all follow-up points (weeks 4, 12, 26 and 52). QALYs for each participant over the trial period will be calculated and will be derived by estimating the area under the curve of the profile generated connecting estimates of HRQoL at each time point using linear interpolation. If necessary, any baseline imbalances in HRQoL at baseline and resulting QALYs will be adjusted using regression methods. QALYs will be derived from utility values from the EQ-5D-5L and HUI3 but the former will be used in the base case analysis.

​​To understand the burden for participants and wider society, healthcare costs will be reported separately from any out-of-pocket expenses and any societal productivity losses. When analysing continuous variables (for example costs, HRQoL scores, QALYs) means and standard deviations will be computed for each trial arm and for comparisons between trial arms, mean differences and 95% parametric confidence intervals will be used. Mean differences in costs and QALYs between study arms will be synthesised using ICERs and to facilitate interpretation, net benefit statistics will be calculated^11^. Uncertainty around net-benefits will be expressed using confidence intervals (if feasible) and cost-effectiveness acceptability curves^12^. Deterministic sensitivity analysis will be used to address uncertainty around study parameters (e.g. unit costs) not participant to sampling variation. Data completeness on resource use and HRQoL will be closely monitored on a regular basis and missing data mechanisms discussed. Multiple imputation will be implemented to handle missing data if a complete case analysis results in substantial loss of information that could bias our results^13^. All analyses will be conducted and reported in accordance with existing good practice guidelines for economic evaluation^14,15^.

# 12. PARTICIPANT EXPERIENCE QUALITATIVE ASSESSMENT

## 12.1 Participant experience

A sample of participants will be invited to join focus groups to be conducted approximately 3, 6 and 12 months after randomisation. These groups are designed to understand the issues participants may have had accessing tinnitus care before the trial, the way they interacted with the trial interventions, any areas of the interventions that could be improved, and finally to discuss how these interventions may be introduced to the NHS. The focus group data will be used to aid interpretation of the quantitative study data and the health economic evaluation, and at the end of the trial will be disseminated alongside the main trial results.

## 12.2 Sampling

The DEFINE Trial Manager will generate a list of participants who have consented to further contact for the focus group. Targeted sampling will be conducted 4 months after the start of recruitment, and then further rounds will be undertaken 3 and 9 months later. Participants will not be selected for more than one focus group timepoint. Where possible the selected sample will be weighted to Office of National Statistics criteria for a nationally representative group based on several variables (age, sex, ethnicity, geographical region), and also balanced across the randomised intervention given, and severity of tinnitus. Investigators will contact those in the sample list via email and/or phone until the required number of participants at each time point is reached.

## 12.3 Focus group consent

Consent for focus group participation and data analysis is included as an optional item on the main DEFINE study consent form.

The transcript data from consenting individuals may additionally be used for publication/media release in journals, newspapers, radio, interviews, TV, social media, websites, newsletters, marketing in print and online. Participants will be provided with a list of the above media types, and asked if willing to consent to use in all or specific dissemination formats.

## 12.4 Focus group design

Focus groups will be semi-structured based on a topic guide. Three focus groups, with 4 participants in each group and lasting up to 60 mins each will be conducted online using Microsoft Teams software. One group will be conducted in the morning, a further in the afternoon, and the final in the evening in an attempt to accommodate a range of individuals. As with sampling, groups will be mixed so that they vary in participant age, sex, ethnicity, geographical location and tinnitus severity. Video recordings will be made using MS teams to record the focus group discussion in its entirety and the transcript will be downloaded following the session.

All participants will be reminded before participation that they will be in an online group situation and accordingly, will be identifiable to the other participants.

A trained facilitator who is part of the DEFINE trial team will coordinate and guide the focus groups.

## 12.5 Focus group topics

A semi-structured topic guide will be used by the facilitator to initially engage participants in key topics and prompt focussed discussions. The topic guide will be designed based on clinical experience of the investigators, and those who deliver CBT and developed the Oto programme. Our PPIE group will also be consulted on topic guide development. The guide will explore:

- barriers to seeking and obtaining advice and treatment for tinnitus
- participant ideas, concerns and expectations about the trial interventions
- the way participants interacted with the trial interventions
- suggested improvements to the delivery or content of the trial interventions
- how these interventions may best be deployed within the NHS

## 12.6 Analysis and outcomes

The transcript files will be downloaded from Microsoft Teams and checked against the original recording for accuracy by SR who will generate an orthographic transcription for thematic analysis. The video will be deleted after checking is complete.

The transcripts will be analysed using an inductive thematic analysis, an open coding approach in which themes and codes are generated from the data. This analysis follows a 6-step process outlined by Braun & Clarke 2006^17^ familiarising oneself with the data (step 1), generating codes (step 2), constructing themes (step 3), reviewing potential themes (step 4), defining and naming themes (step 5), and producing the report (step 6). The approach will follow the adapted process for group analysis as applied by Campbell et al. (2021^18^). The initial familiarisation phase will involve two researchers jointly reviewing the focus group data to discuss and establish an overall sense of the data. The researchers will then read the transcripts individually before working jointly again to generate codes and themes. The themes will then be reviewed and defined, before producing the report.

# 13 DATA MANAGEMENT

## 13.1 Source Data

Source data are where data are first recorded, and from which participants’ survey data are obtained. These include, but are not limited to, medical records, pharmacy records, participant surveys, and correspondence.

EDC entries will be considered source data if the EDC is the site of the original recording. All documents will be stored securely and safely in confidential conditions.

## 13.2 Access to Data

Data will be entered into a validated EDC platform. Direct access to the EDC platform will be granted to authorised representatives from the Sponsor and Lindus Health to allow trial-related monitoring and/or audits to ensure compliance with regulations. Access to this system will be strictly on a need to know basis and the system will be on a secure server. To ensure data transparency, the trial will be registered on a publicly available database for clinical studies.

## 13.3 Data Handling and Record Keeping

The data management will be run in accordance with Lindus Health SOPs, which are fully compliant with Good Clinical Practice (GCP), GDPR and the Data Protection Act 2018. Research data will be de-identified upon collection. Identifiable information will be stored separately from the research data.

A unique trial specific number and/or code in the database will identify the participants.

An online secure data entry system designed to collect sensitive data, such as participant and Study Partner contact details, will be used. All identifiable participant data is encrypted using the Advanced Encryption Standard. The participant portal will also manage online ePRO.

Informed consent (e-consent) will be completed using worksheet templates, which will be retained in a secure area with restricted access. The remaining data, including reconfirmation of consent and records of eligibility confirmation will be collected directly onto the EDC platform, therefore no further source will be available.

Identifiable participant data will be kept for 12 months beyond the end of the study. Non-identifiable participant data will be maintained for a minimum of ten years unless otherwise required to comply with legislation or regulation and reviewed on an annual basis.

# 14. QUALITY ASSURANCE PROCEDURES

The study may be monitored, or audited in accordance with the current approved protocol, GCP, relevant regulations and standard operating procedures.

## 14.1 Risk Assessment

The risk assessment and categorization tool will be used to identify and evaluate potential risks to participant safety, data quality, and study integrity, and to determine the appropriate risk mitigation strategies. This tool will include an assessment of various aspects of the study, such as participant recruitment, data collection, and intervention administration. The tool will be reviewed and updated throughout the study to ensure ongoing risk management.

## 14.2 Study Monitoring

Monitors may verify that the clinical study is conducted, and data are generated, documented and reported in compliance with the protocol, GCP and the applicable regulatory requirements. Specifics will be detailed in the Trial Monitoring Plan.

## 14.3 Study Committees

A Trial Steering Committee (TSC) and Trial Management Group (TMG) will be appointed. The responsibilities of each group are as follows:

TSC- to review the safety data and to review and monitor the accruing data to ensure the rights, safety and wellbeing of the trial participants. They will make recommendations about how the study is operating and any ethical or safety issues. This Committee will also take on the role of a Trial Steering Committee, and so act as a single oversight committee meeting at least bi-annually.

Trial Management Group (TMG)- is responsible for the day-to-day running of the trial, including monitoring all aspects of the trial and ensuring that the protocol is being adhered to. It will include the Lindus Health Clinical Operations Team, Medical Lead and Product Manager working closely with the CI and Sponsor and will meet weekly in the first instance. After the initial weekly meetings, the TMG will meet on a regular basis, such as monthly or bi-monthly, to discuss study progress and address any issues that may arise.

# 15. PROTOCOL DEVIATIONS

A study related deviation is a departure from the ethically approved study protocol or other study document or process (e.g. consent process or administration of study intervention) or from Good Clinical Practice (GCP) or any applicable regulatory requirements. Any deviations from the protocol will be documented, assessed as to whether it is a potential Serious Breach (according to the definition in Section 15) and filed in the electronic Trial Master File (eTMF), according to Lindus Health SOPs. All protocol deviations will be recorded in a Protocol Deviation Tracker and discussed with the CI and Sponsor on a regular basis during the course of the study.

# 16. SERIOUS BREACHES

A “serious breach” is a breach of the protocol or of the conditions or principles of Good Clinical

Practice which is likely to affect to a significant degree

(a) the safety or physical or mental integrity of the trial participants; or

(b) the scientific value of the research.

In the event that a serious breach is suspected the Sponsor must be contacted within one working day. In collaboration with the CI, the serious breach will be reviewed by the Sponsor and, if appropriate, the Sponsor will report it to the approving REC committee within seven calendar days.

# 17. ETHICAL AND REGULATORY CONSIDERATIONS

## 17.1 Declaration of Helsinki

The Investigator will ensure that this study is conducted in accordance with the principles of the Declaration of Helsinki (2013).

## 17.2 Guidelines for Good Clinical Practice

The Investigator will ensure that this trial is conducted in accordance with relevant regulations and with Good Clinical Practice.

## 17.3 Approvals

The protocol, informed consent form, participant information sheet and any proposed advertising material will be submitted to an appropriate Research Ethics Committee (REC) and HRA for written approval. The Investigator will submit and, where necessary, obtain approval from the above parties for all substantial amendments to the original approved documents. Substantial amendments are those that have a significant impact on the safety or welfare of the participants, the scientific value of the study, or the conduct or management of the study.

## 17.4 Reporting

An Annual Progress Report will be submitted once a year to the REC, HRA (where required) and Sponsor. In addition, an End of Trial notification and final report will be submitted to the REC and Sponsor.

## 17.5 Participant Confidentiality

The trial staff will ensure that the participants’ anonymity is maintained. The participants will be identified only by a participant ID number on all trial documents and in the electronic clinical database. All data will be stored securely on an electronic study database and will comply with the General Data Protection Regulation (GDPR) and Data Protection Act 2018, which require data to be de-identified as soon as it is practical to do so. The study database will be managed according to Standard Operating Procedures maintained by Lindus Health. Access rights to data and applications software will be clearly defined and staff authorised to access personal data will be formally notified in writing of the permissible scope of their access. Data access will be limited to specific members of the research team (trained in data protection policy) including the chief investigator (as study guarantor), and engineers. For each database application, system users will be given a valid user system account name (username ID), and a password known only to that user to prevent unauthorised use of systems. All data will be entered into the database through a reliably encrypted gateway.

## 17.6 Expenses and Benefits

Participants in the smartphone app delivered arm will receive free access to the intervention for the 12 months of the study. Those in the control group will receive a free subscription to the Oto tinnitus programme at the end of the 12 month trial period.

## 17.7 Patient and Public Involvement and Engagement (PPIE)

The Lindus Health Patient Advisory Board has provided substantial input to the protocol and the overall study design. A study PPIE group has been formed and provided input into patient facing materials including the study design, patient information sheet, information video and informed consent form. The EDC platform has undergone user testing to ensure that it is user friendly and the language is understood.

# 18. FINANCE AND INSURANCE

## 18.1 Funding

This study will be funded by Innovate UK (UKRI) (70%) and Oto Health (30%) and conducted by Lindus Health.

## 18.2 Insurance

The Sponsor has a specialist insurance policy in place for this study which would operate in the event of any participant suffering harm as a result of their involvement in the research.

## 18.3 Contractual Arrangements

Appropriate contractual arrangements with third parties will include agreements covering the provision of goods and services, data sharing and management, and intellectual property rights, among other matters. These contracts will be established to ensure that all parties are aware of their roles and responsibilities and to protect the interests of the study and its stakeholders.

# 19. PUBLICATION POLICY

The Chief Investigator will lead the collaborative drafting of the manuscripts, abstracts, press releases and any other publications arising from the study. Authors will acknowledge that the study was delivered via a clinical trial agreement between Oto Health and Lindus Health. Authorship will be determined in accordance with the International Committee Medical Journal Editors guidelines and other contributors will be acknowledged.

The study results will be published in an open-access journal or repository, ensuring that the research findings are accessible to a wide audience, including healthcare professionals, researchers, and the public.

No publication or disclosure of study results will be permitted except under the terms and conditions of a separate written agreement between Oto Health and the CI.

# 20. ARCHIVING

Study materials will be archived according to Lindus Health SOPs for a minimum of ten years, personal data will be retained for a minimum of 12 months, compliant with applicable legislation and trial regulations.

# 21. REFERENCES

1. Biswas, R., A. Lugo, M. Akeroyd, W. Schlee, S. Gallus, and D. Hall. 2022. “Tinnitus prevalence in Europe: a multi-country cross-sectional population study.” *The Lancet Regional Health-Europe* 1, no. 12 (Jan): 100250. DOI: 10.1016/j.lanepe.2021.100250.
2. Jarach, C. M., A. Lugo, M. Scala, P. A. van den Brandt, C. R. Cederroth, A. Odone, W. Garavello, W. Schlee, B. Langguth, and S. Gallus. 2022. “Global prevalence and incidence of tinnitus: A systematic review and meta-analysis.” *JAMA Neurology*, (Sep). DOI: 10.1001/jamaneurol.2022.2189.
3. Landry, E. C., X. C. Sandoval, C. N. Simeone, G. Tidball, J. Lea, and B. D. Westerberg. 2020. “Systematic review and network meta-analysis of cognitive and/or behavioral therapies (CBT) for tinnitus.” *Otology & Neurotology* 41, no. 2 (Feb): 153-66. DOI: 10.1097/MAO.0000000000002472.
4. National Institute for Health and Care Excellence (NICE). 2020. “Tinnitus: assessment and management.” Tinnitus: assessment and management.
5. Pandit, J. A., J. M. Radin, G. Quer, and E. J. Topol. 2022. “Smartphone apps in the COVID-19 pandemic.” Nature Biotechnology 40:1013-1022. DOI: 10.1038/s41587-022-01350-x.
6. Weisel, K. K., L. M. Fuhrmann, M. Berking, H. Baumeister, P. Cuijpers, and D. D. Ebert. 2019. “Standalone smartphone apps for mental health—a systematic review and meta-analysis.” NPJ Digital Medicine 118 (Dec). DOI: 10.1038/s41746-019-0188-8.
7. Nagaraj, M. K., and P. Prabhu. 2020. “Internet/smartphone-based applications for the treatment of tinnitus: a systematic review.” European Archives of Oto-Rhino-Laryngology 277 (Jun): 649-657. DOI: 10.1007/s00405-019-05743-8.
8. Drummond MF, Sculpher MJ, Claxton K, Stoddart GL, Torrance GW. Methods for

the Economic Evaluation of Health Care Programmes [Internet]. York Research

Database. Oxford: Oxford University Press; 2015

1. Herdman, M., C. Gudex, A. Lloyd, M. Janssen, P. Kind, D. Parkin, G. Bonsel, and X.

Badia. Development and preliminary testing of the new five-level version of

EQ-5D (EQ-5D-5L). Qual Life Res, 2011. 20(10): 1727-36.

1. National Institute for Health and Care Excellence. NICE health technology

evaluations: the manual. 2022, National Institute for Health and Care

Excellence: London.

1. Stinnett, A.A. and J. Mullahy. Net health benefits: a new framework for the analysis

of uncertainty in cost-effectiveness analysis. Med Decis Making, 1998. 18(2 Suppl):

S68-80.

1. Fenwick, E., K. Claxton, and M. Sculpher. Representing uncertainty: the role of

cost-effectiveness acceptability curves. Health Econ, 2001. 10(8): 779-87.

1. Faria, R., M. Gomes, D. Epstein, and I.R. White. A guide to handling missing data in

cost-effectiveness analysis conducted within randomised controlled trials. Pharmacoeconomics, 2014. 32(12): 1157-70.

1. Husereau, D., Drummond, M., Augustovski, F., de Bekker-Grob, E., Briggs, A.H., Carswell, C., Caulley, L., Chaiyakunapruk, N., Greenberg, D., Loder, E., Mauskopf, J.,

Mullins, C.D., Petrou, S., Pwu, R.F., Staniszewska, S., and Force, C.I.G.R.P.T.,

Consolidated Health Economic Evaluation Reporting Standards 2022 (CHEERS

2022) Statement: Updated Reporting Guidance for Health Economic Evaluations.

Value Health, 2022. 25(1): p. 3-9.

1. Ramsey, S.D., Willke, R.J., Glick, H., Reed, S.D., Augustovski, F., Jonsson, B., Briggs, A., and Sullivan, S.D., Cost-effectiveness analysis alongside clinical trials II-An ISPOR Good Research Practices Task Force report. Value Health, 2015. 18(2): p. 161-72.
2. Stockdale, D., D. McFerran, P. Brazier, C. Prichard, T. Kay, C. Dowrick, and D. J. Hoare. 2017. “Stockdale D, McFerran D, Brazier P, Pritchard C, Kay T, Dowrick C, Hoare DJ. An economic evaluation of the healthcare cost of tinnitus management in the UK.” BMC Health Services Research 17, no. 1 (Dec): 1-9. DOI: 10.1186/s12913-017-2527-2.
3. Braun V, Clarke V. Using thematic analysis in psychology. Qualitative Research in Psychology. 2006
4. Campbell, K., Orr, E., Durepos, P., Nguyen, L., Li, L., Whitmore, C., Gehrke, P., Graham, L., Jack, S. Reflexive thematic analysis for applied qualitative health research. Qualitative Report (2021) 26(6) 2011-2028
5. Beukes et al. Audiologist-Guided Internet-Based Cognitive Behavior Therapy for Adults With Tinnitus in the United Kingdom: A Randomized Controlled Trial; 2018.

#

# 22. APPENDIX 1 – TRIAL FLOW-CHART

| **Recruitment**  Primary care, social media, patient communities |
| --- |

↓

| **Prescreening and Registration**  Potential participants directed to LH website. Registered in the LH system. PIS, ICF, video emailed, screening call booked |
| --- |

↓

| **Enrolment**  Potential participants checked against I/E criteria, consent confirmed, randomised to study arm. |
| --- |

↓

| **Sponsor, Participant, GP notified**  Details of randomised participants shared and enrolment confirmed |
| --- |

↓

| **Intervention Arm**  Onboarded to the Oto Tinnitus Programme for 6W | **←** | **Treatment Phase Begins**  Participants enrolled on schedule of assessments and baseline collected | **→** | **Control Arm**  Therapist delivered CBT by a trained specialist |
| --- | --- | --- | --- | --- |

↓

| **Month 1 Follow Up**  Remote contact for outcome measurements |
| --- |

↓

| **Month 3 Follow Up**  Remote contact for outcome measurements |
| --- |

↓

| **Month 6 Follow Up**  Remote contact for outcome measurements |
| --- |

↓

| **Month 12 Follow Up**  Remote contact for outcome measurements |
| --- |

↓

| **End of Trial** |
| --- |

# 23. APPENDIX 2 - SCHEDULE OF EVENTS TABLE

| **Procedure** | **Pre-screener**  Week -*N* | **Screening**  Week 1, Day 0 | **Randomisation**  Day 0 | **Baseline**  Week 1, Day 1 (+ 7 days) | **Oto App starts for 6wks / consultation for CBT booked**    Day 1 | **Month 1 Follow Up**  Week 4, Day 1 (+/- 7 days) | **Month 3 Follow Up**  Week 12, Day 1 (+/- 14 days) | **Month 6 Follow Up**  Week 24, Day 1 (+/- 14 days) | **Month 12 Follow Up**  Week 52, Day 1 (+/- 28 days) |
| --- | --- | --- | --- | --- | --- | --- | --- | --- | --- |
| Informed Consent | ✓ | ✓ |  |  |  |  |  |  |  |
| Eligibility assessment | ✓ | ✓ |  |  |  |  |  |  |  |
| Medical history | ✓ | ✓ |  |  |  |  |  |  |  |
| Demographics |  | ✓ |  |  |  |  |  |  |  |
| Tinnitus Background Survey |  |  |  | ✓ |  |  |  |  |  |
| Tinnitus Functional Index (TFI) |  |  |  | ✓ |  | ✓ | ✓ | ✓ | ✓ |
| EQ-5D-5L |  |  |  | ✓ |  |  | ✓ | ✓ | ✓ |
| HUI3 |  |  |  | ✓ |  |  | ✓ | ✓ | ✓ |
| Resource Use Questionnaire |  |  |  |  |  | ✓ | ✓ | ✓ | ✓ |
| System Usability Scale |  |  |  |  |  |  | ✓ |  |  |
| AE review |  |  |  | ✓ |  | ✓ | ✓ |  |  |
| Study Experience Questionnaire |  |  |  |  |  |  | ✓ | ✓ | ✓ |
| Adherence Data collected (eCRF) |  |  |  |  |  | ✓ | ✓ | ✓ | ✓ |

#

#

#

#

#

#

#

# 24. APPENDIX 3 – CONTROL ARM INTERVENTION SPECIFICATION

Control arm intervention – specification

The standard care intervention was developed based on relevant guidance (BSA Practice Guidance Tinnitus in adults 2021 and NICE guidelines (NG-155)) and via consultation with clinicians providing NHS tinnitus therapy.

New assessment (approximately 60 minutes) to include:

1. Information gathering and education

- Detailed audiological history to include onset, progression, nature and impact of tinnitus at home/work/social, on sleep, mental health and quality of life.
- Explanation of tinnitus, tailored to the patients’ needs and understanding, usually based on a neurophysiological model (Jastreboff PJ, 1990) or cognitive behavioural model (McKenna et al 2014)
- Explanation of how hearing loss may influence tinnitus awareness
- Discussion of options for treatment/ rehabilitation

Questionnaires should be administered as appropriate to assess tinnitus handicap, anxiety and depression in order to establish the functional impact of tinnitus and guide appropriate management, and if appropriate onward referral to other specialties. Example questionnaires for this purpose include: Tinnitus Handicap Inventory; Tinnitus Functional Index; Hospital Anxiety and Depression Scale; Insomnia Severity Index

2. Individual Management Plan

These should be formulated individually, agreed with the participant and may include:

- Relaxation Therapy: Exercises, apps, refer to physio where indicated/available
- CBT: where available. Clinicians may undertake CBT if they have appropriate training and experience to do so. Others may suggest CBT exercises as part of therapy where appropriate, or signpost to other local services, where these exist.
- Signposting for equipment to facilitate sound enrichment and relaxation
  - - Sound generators with/ without pillow speakers
    - Ear level or in the ear sound generators
    - Relaxation CD
- Recommendation for hearing aid fitting where hearing loss is present and discussion of combination hearing aid/ tinnitus maskers where relevant
- Information leaflets which include websites with more information about tinnitus and products available
- Tinnitus UK
- Royal National Institute for the Deaf
- Treatment for anxiety/depression: signpost to local wellbeing service or to GP for antidepressant medication

Follow-up appointments if indicated (30 min routine/ 45 min complex) to include:

- Revision of history of changes
- Discussion/ documentation of progress and outcome of treatment plan
- Onward referral if appropriate
- Further follow-ups can be arranged as required and according to departmental policy.

At any stage in the intervention the therapist may consider onward referral if appropriate and after discussion with the participant

This could include the following:

- Audiology: hearing aid assessment and fitting, ear level sound generator fitting/ combi device fitting or further diagnostic tests
- GP: to request counselling or a formal psychological or psychiatric referral; medication review

o Where a patient has significant anxiety or depression and is judged to be at significant risk of self-harm, the GP should be contacted immediately by phone. The patient should be made aware of this and encouraged to see their GP urgently.

o If a patient has significant anxiety and depression but is not suicidal, then discuss a local referral with their GP, or self-refer for psychology support via Improving Access to Psychological Therapies.

- ENT: further tinnitus investigations, vestibular assessment
- Physiotherapy: relaxation exercises.

# 25. APPENDIX 4 – AMENDMENT HISTORY

| Amendment Number | Protocol Version | Date Issued | Author(s) of changes | Date changes made |
| --- | --- | --- | --- | --- |
|  |  |  |  |  |
|  |  |  |  |  |

Lists details of all protocol amendments whenever a new version of the protocol is produced.

Protocol amendments must be submitted to the Sponsor for approval prior to submission to the REC committee and HRA (where required)
