# Supplementary material for: Digital thErapy For Improved tiNnitus carE Study (DEFINE): Protocol for a randomised controlled trial
Source: PLoS One. 2024 Jan 5;19(1):e0292562. doi: 10.1371/journal.pone.0292562 (PMC10769067; doi:10.1371/journal.pone.0292562)
Supplement: S2 File — ’Intervention details for the control arm of standard tinnitus therapy’. (DOCX) [file pone.0292562.s003.docx]

Control arm intervention – specification

The standard care intervention was developed based on relevant guidance (BSA Practice Guidance Tinnitus in adults 2021 and NICE guidelines (NG-155)) and via consultation with clinicians providing NHS tinnitus therapy.

## New assessment (approximately 60 minutes) to include:

1. Information gathering and education

- Detailed audiological history to include onset, progression, nature and impact of tinnitus at home/work/social, on sleep, mental health and quality of life.
- Explanation of tinnitus, tailored to the patients’ needs and understanding, usually based on a neurophysiological model (Jastreboff PJ, 1990) or cognitive behavioural model (McKenna et al 2014)
- Explanation of how hearing loss may influence tinnitus awareness
- Discussion of options for treatment/ rehabilitation

Questionnaires should be administered as appropriate to assess tinnitus handicap, anxiety and depression in order to establish the functional impact of tinnitus and guide appropriate management, and if appropriate onward referral to other specialties. Example questionnaires for this purpose include: Tinnitus Handicap Inventory; Tinnitus Functional Index; Hospital Anxiety and Depression Scale; Insomnia Severity Index

1. Individual Management Plan

These should be formulated individually, agreed with the patient and may include:

- Relaxation Therapy: Exercises, apps, refer to physio where indicated/available
- CBT: where available. Clinicians may undertake CBT if they have appropriate training and experience to do so. Others may suggest CBT exercises as part of therapy where appropriate, or signpost to other local services, where these exist.
- Signposting for equipment to facilitate sound enrichment and relaxation
  - - Sound generators with/ without pillow speakers
    - Ear level or in the ear sound generators
    - Relaxation CD
- Recommendation for hearing aid fitting where hearing loss is present and discussion of combination hearing aid/ tinnitus maskers where relevant
- Information leaflets which include websites with more information about tinnitus and products available
  - - Tinnitus UK
    - Royal National Institute for the Deaf
- Treatment for anxiety/depression: signpost to local wellbeing service or to GP for antidepressant medication

## Follow-up appointments if indicated (30 min routine/ 45 min complex) to include:

- Revision of history of changes
- Discussion/ documentation of progress and outcome of treatment plan
- Onward referral if appropriate
- Further follow-ups can be arranged as required and according to departmental policy.

## At any stage in the intervention the therapist may consider onward referral if appropriate and after discussion with the participant

This could include the following:

- Audiology: hearing aid assessment and fitting, ear level sound generator fitting/ combi device fitting or further diagnostic tests
- GP: to request counselling or a formal psychological or psychiatric referral; medication review
  - Where a patient has significant anxiety or depression and is judged to be at significant risk of self-harm, the GP should be contacted immediately by phone. The patient should be made aware of this and encouraged to see their GP urgently.
  - If a patient has significant anxiety and depression but is not suicidal, then discuss a local referral with their GP, or self-refer for psychology support via Improving Access to Psychological Therapies.
- ENT: further tinnitus investigations, vestibular assessment
- Physiotherapy: relaxation exercises.

# References

BSA Practice Guidance Tinnitus in adults 2021 (<https://www.thebsa.org.uk/wp-content/uploads/2021/12/OD104-95-BSA-practice-guidance-Tinnitus-in-adults-Publication-Nov-2021-1.pdf>)

Tinnitus Assessment and Management NICE guidelines (NG-155) 2020 Available here: [Overview | Tinnitus: assessment and management | Guidance | NICE](https://www.nice.org.uk/guidance/ng155)

Jastreboff PJ. Phantom auditory perception (tinnitus): mechanisms of generation and perception. Neurosci Res. 1990 Aug;8(4):221-54. doi: 10.1016/0168-0102(90)90031-9. PMID: 2175858.

McKenna L, Handscomb L, Hoare DJ, Hall DA. A scientific cognitive-behavioral model of tinnitus: novel conceptualizations of tinnitus distress. Front Neurol. 2014 Oct 6;5:196. doi: 10.3389/fneur.2014.00196. PMID: 25339938; PMCID: PMC4186305
