# Supplementary material for: Digital thErapy For Improved tiNnitus carE Study (DEFINE): Protocol for a randomised controlled trial
Source: PLoS One. 2024 Jan 5;19(1):e0292562. doi: 10.1371/journal.pone.0292562 (PMC10769067; doi:10.1371/journal.pone.0292562)
Supplement: S3 File — ‘Patient information sheet for the trial’. (PDF) [file pone.0292562.s004.pdf]

PARTICIPANT INFORMATION SHEET (PIS)

Study title: Digital thErapy For Improved tiNnitus carE (DEFINE)
Sponsor: Oto Health Ltd.
Study conducted by: Lindus Health
Chief Investigator: Dr. Matthew Smith
Trial Contact Details: define@lindushealth.com

You have been invited to take part in the DEFINE trial because we understand that you have suffered with tinnitus for at least three months. This trial aims to investigate whether the Oto Tinnitus Programme can reduce the distress caused by tinnitus. This programme is app-delivered tinnitus therapy based on the principles of Cognitive Behavioural Therapy (CBT), a well-established psychological treatment.

Participation in this trial is entirely voluntary. Before you decide whether you would like to take part, it is important for you to understand why the research is taking place and what it would involve for you. A member of the research team will go through this information sheet with you and answer any questions you have.

Please take time to read the following information carefully. You may talk to others about the study if you wish.

Take your time to decide whether or not you wish to take part. If you have any questions, or if you would like more information about the study, please feel free to get in touch with the trial team:
Freephone: 0800 058 4496 Email address: define@lindushealth.com

Digital thErapy For Improved tiNnitus carE Study: The *DEFINE* Trial

PARTICIPANT INFORMATION SHEET (PIS)

What is the purpose of this study?

The purpose of this study is to find out whether the Oto Tinnitus Programme is no worse at reducing the negative effects of tinnitus as one-to-one tinnitus therapy incorporating Cognitive Behavioural Therapy (CBT). The trial also aims to understand the financial implications of both approaches. It is important to note that like conventional therapy, the app is not a treatment or cure for tinnitus. Rather, it is designed to support your overall well-being and teach you techniques to help you live with tinnitus.

What is the Oto Tinnitus Programme?

The Oto Tinnitus Programme is a digital approach to tinnitus management, delivered as a self-paced smartphone app. It is based on CBT, which is talking therapy that aims to identify and change negative thoughts and behaviours. The app also includes education, mindfulness and physical therapy (physical stretches and exercises).

The Oto Tinnitus Programme is delivered to patients using the Oto app. This app provides modules to guide and support you, as well as useful information about managing your tinnitus. The app includes several different types of therapy to help you achieve your goals. You will be required to download the app and will be asked to sign up to the app's services.

Please visit the Oto website for more detailed information: https://www.joinoto.com/

What's involved?

After consenting to take part in the study and taking a short hearing test (guided by a member of the research team), you will be randomly allocated to one of two groups. One group will follow the Oto Tinnitus Programme which takes up to six weeks to complete, while the other will receive standard CBT delivered remotely by a trained audiologist, hearing therapist or psychologist. This standard CBT will initially be one session, with further sessions available (up to a maximum of six) if agreed by the psychologist and participant.

You will remain in the same group for the duration of the study, and will therefore have the same treatment throughout.

The entire study will be conducted remotely, without in-person visits. Youwill complete a telephone/video call with a trained member of the trial team to confirm eligibility and complete informed consent.

At the beginning of the study, and again after one, three, six and twelve months, you will be asked to complete questionnaires about your tinnitus, quality of life, and any healthcare visits and costs you may have undertaken. These questionnaires will be completed electronically through the Lindus Health electronic questionnaire, using your smartphone, tablet or computer.

Can I take part?

We are inviting individuals who are experiencing tinnitus, aged 18 or above, to participate in this study. We welcome people with or without hearing aids. A member of the study team will check whether you are suitable for the study based on the study eligibility criteria and the data you provide.

Do I have to take part?

Participation in the study is entirely voluntary, and it is up to you to decide if you want to take part. If you do decide to take part, you will be asked to read this Participant Information Sheet (PIS) and then sign the Informed Consent Form (ICF). If you do not want to take part, your standard tinnitus care will not be impacted.

You are welcome to discuss the study, the PIS and the ICF with the study team, and will be given every opportunity to ask questions. After consenting, you are still free to withdraw at any time, without giving a reason, and without your medical care being affected. Your GP or a study investigator also has the authority to withdraw you from the study if they feel it is appropriate.

What will happen to me if I take part?

If you agree to take part, you will be asked to complete a short online form to see if you are eligible. If you meet the eligibility criteria, this PIS will be emailed to you and you will be asked to book a telephone or video call with a member of the study team.

On this call, a member of the study team will assist you in conducting a short hearing test using a free third party phone application. To do this test you will need to have a smartphone with headphones and take the call in a quiet place.

Informed consent

During the call, a member of the study team will explain what the study involves and the potential risks and benefits of taking part. They will also answer any questions you may have. You will be asked a few questions about your health to further assess your eligibility for the study. After this discussion, if you are still willing and eligible to take part, you will be asked to provide informed consent by electronically signing a consent form online, a copy of which will be emailed to you. The study team member will also

sign this form, and you will be given a fully signed copy for your records.

What treatment will I get?

You will be randomly allocated to one of two groups, using a computer-generated system. Half of the participants will be allocated to the standard tinnitus therapy group, and half to the Oto Tinnitus Programme. Group assignments cannot be changed after randomisation.

- **Standard tinnitus therapy group:**
    - Receiving therapist-delivered CBT; This will consist of a minimum of 1 session up with further sessions at the discretion of yourself and therapist up to a maximum of 6.
- **Oto Tinnitus Programme group:**
    - Receiving the Oto Tinnitus Programme; The programme lasts approximately 6 weeks, using the app 4-5 times per week.

Once your eligibility is confirmed, the study team will let you know which group you have been allocated to.

If you are allocated to the Oto Tinnitus Programme, your access will start immediately. If you are allocated to the standard CBT group, your first session with the therapist will be booked, to start within a week.

Neither you nor the study team member can choose which group you will be allocated to. You will remain in the same group throughout the trial.

Lindus Health electronic questionnaire

You will need to set up the Oto App (if on the Oto Tinnitus Programme) on your smartphone and complete the Lindus Health electronic questionnaires throughout the study. Sometimes the same information will be needed on both systems, which means you may have to enter the data twice.

During your initial phone call, the Lindus Health study team will go through the process of setting up all of the required software. The Lindus Health team and Oto team will be available to help if any problems arise during the study.

1. Getting started

Once you have been allocated to a group, you will be asked to complete baseline assessments.

You will need to:

- Complete the questionnaires. This will include:
    - A questionnaire about your tinnitus

- Questionnaires about your quality of life as it relates to your health
- A questionnaire about the contact you have had with the health service and any other products or treatments you have tried for your tinnitus

2. Questionnaires Throughout Trial(Months 1, 3, 6 and 12)

At one, three, six and 12 months, you will need to provide the following information in your online questionnaire:

- Any side effects or changes in tinnitus that you have experienced, since starting the trial.
- A questionnaire about the contact you have had with healthcare services and any other treatments or costs in relation to your tinnitus.
- A questionnaire about your tinnitus
- Questionnaires about your quality of life as it relates to your health
- A questionnaire about the contact you have had with the health service and any other products or treatments you have tried for your tinnitus

3. Focus Groups / Semi-Structured Interviews (Months 3 and 6)

As part of the study, we will be conducting focus group discussions and semi-structured interviews at months 3 and 6 to gather insights and experiences from participants. These are voluntary and we may invite you to participate, but you can decline involvement in these additional groups without affecting your treatment or position in the study. These discussions will be recorded using MS Teams for analysis purposes.

Expenses and payments

You can receive £40 as financial compensation for taking part in this research study. The breakdown of payment is as follows:

- Month 1 and Month 3 surveys - £5 each
- Month 6 and Month 12 surveys - £10 each
- Quality Surveys - £10

Access to the Oto smartphone app will be provided, free of charge, for anyone in the Oto intervention group. Anyone in the standard tinnitus therapy group will receive access to standard care, , provided one-to-one by an audiologist/hearing therapist.

For those allocated to the standard tinnitus therapy group, you will also be offered the Oto smartphone app free of use for 12 months at the end of the trial.

What are the possible benefits of taking part?

Taking part in this study will allow you to receive treatment for your tinnitus without a significant wait. Participants in the standard tinnitus therapy group will receive rapid access to one-to-one therapy, rather than typical waiting times of more than 12 months from GP referral. Participants in the intervention group will receive immediate access to therapies through the Oto app. The Oto App and one-to-one delivered tinnitus therapy are free of charge.

In addition, information gathered from this study can be used to improve the management and treatment of tinnitus for other people in the future.

What are the possible disadvantages and risks of taking part?

No significant risks are anticipated, as the Oto Health Tinnitus Programme is based on cognitive behavioural therapy (CBT). There are no known side effects associated with CBT, but participants will be made aware that they need to seek medical attention if their tinnitus worsens. Completing the questionnaires will take time, but these have been designed to be as short as possible and easy to access online.

What if I start to experience side effects or a medical event?

Please complete the scheduled questionnaire, where you can report the side effects. You can also contact the study team to report any information you think we should be aware of via phone or email. **NOTE: Please call 999 if you are having a medical or mental health emergency.** All other healthcare services are available to you as usual.

What happens when the research study stops?

When the study ends, you will continue to be treated by your GP under standard routine care.

Free access to the app will continue for participants in the OTO arm, for those in the standard CBT group, you will also be offered the Oto Tinnitus app at the end of the trial for 12 months, free of charge. You will have finished the trial at this stage so you will not be followed up by the research team.

After the end of the trial, we will not collect any further trial data via the app. The data collected from the app is outlined below: 'How will we use information about you?'

Will my taking part in the study be kept confidential?

People allowed to look at your health information will be limited, to include the research team, the sponsor and the regulatory authorities who check that the trial is being carried out to legal and ethical standards. On joining the study participants will be given a unique ID number and this will be used to

label their study information so your name does not appear with your questionnaire or other results. Your identity will not be revealed in the results or any publications.

If you have further questions, please see the 'Frequently asked questions' or email define@lindushealth.com.

What if relevant new information becomes available?

Sometimes new information becomes available about the product used in a study while the trial is ongoing. If new information becomes available, we will notify you. The relevant ethical authorities will also be informed and the study may be stopped or changed if appropriate. If the information sheet changes significantly, you will be asked to read the new information sheet and sign another consent form. You will have the opportunity to discuss these changes with the Lindus Health team and ask any questions. You will be able to withdraw from the study at any stage.

What will happen if I don't want to carry on with the study?

You can withdraw from the study at any time, without giving a reason, and this will not affect the standard of care you receive or your legal rights. If you withdraw from the study, you are advised to contact your GP.

If you decide to withdraw, or are no longer able to consent, we will still use all the data collected up to the time of your withdrawal. This means that all the questionnaires that you have submitted will be analysed.

What if there is a problem?

Complaints

If you have any concerns about any aspect of this study, you can contact define@lindushealth.com, and any complaint, or any possible harm you might suffer, will be addressed.

Am I covered by insurance?

In the unlikely event that you become ill or are injured as a result of taking part in this study, you will be covered by insurance held by the Sponsor. Compensation will be provided for any injury caused by taking part in this study, in accordance with guidelines of the Association of the British HealthTechIndustries (ABHI). We will pay compensation where the injury likely resulted from:

- A product being administered as part of the trial protocol; or

- Any test or procedure you received as part of the trial.

Any payment would be without legal commitment (please enquire if you would like more information on this). The Sponsor would not be bound by these guidelines to pay compensation where:

- The injury resulted from a drug or procedure outside the study; or
- The study procedures were not followed.

In the unlikely event that something does go wrong and you are harmed during the research due to someone's negligence, then you may have grounds for legal action for compensation. In this case, you may have to pay your legal costs. If you wish to make a claim against this insurance you should talk to the Lindus Health team.

This study should not affect your health, but if you have private insurance you should check with your insurance company before agreeing to take part in the study, to ensure that your participation will not affect your medical insurance.

How will we use information about you?

We will use information from you, your medical records and your GP for this research project.

This information will include your:

- Initials
- Name
- Date of birth and/or age
- Contact details
- Medical history

This information will be used to conduct the research, or to check your records to make sure that the research is being done properly. People who do not need to know who you are will not be able to see your name or contact details. Your data will have a code number instead. We will keep all information about you safe and secure. You will also consent to share your name and contact details with third parties. These third parties will be healthcare providers who can supply the study team with additional healthcare data relevant to the trial. This data will only be shared for the duration of the study.

Once the study is finished, we will keep some of the data so we can check the results. We will write our reports in a way that prevents all participants from being identified.

The Oto Tinnitus App collects identifiable information about you, including your age, country and usage data. This information will not be shared outside of the Oto team, and access to this data is limited to those who need it. Please see the Oto Privacy Policy for further details: https://www.joinoto.com/privacy-policy.

What are your choices about how your information is used?

You can stop taking part in the study at any time, without giving a reason. In this case, the information collected up until that point will still be used.

We need to manage your records in specific ways for the research to be reliable. This means that we won't be able to let you see or change the data we hold about you.

Where can you find out more about how your information is used?

You can find out more about how we use your information from the following places:

- Online, at www.hra.nhs.uk/information-about-patients/
- Our leaflet available from www.hra.nhs.uk/patientdataandresearch
- By asking one of the research team
- By sending an email to: research@joinoto.com
- By ringing us on 020 8609 4690.

Other information about your data

Your data will be handled, processed, stored and destroyed in accordance with the Data Protection Act of 2018. We will keep identifiable information about you for up to 12 months after the trial has finished. This doesn't include any research documents with personal information, such as consent forms, which will be held securely for 10 years after the end of the study.

Lindus Health may also retain personal data for business improvement purposes. For example, use of behavioural data to make feature improvements to the Electronic Data Capture platform.

Involvement of the General Practitioner/family doctor GP

Your GP will be informed about your participation in the study. Your GP will be contacted as a safety precaution, and we advise that you contact your GP if you experience any medical problems during or shortly after the study period. We may also ask your GP for confirmation of your medical history. It is important for the Lindus Health team to know about your medical history so that you are not put at any unnecessary risk.

What will happen to the results of the research study?

The results and findings may be published in scientific papers and presented at meetings. Your identity will not be disclosed in any of these, nor will any information that could identify you as a participant in this study. If you wish, we can notify you if an article based on the results of this study is published. We will also release a plain English summary or the trial results.

Who is organising and funding the research?

This study is sponsored and organised by Oto Health and conducted by Lindus Health. The Chief Investigator is Dr. Matthew Smith.

Who has reviewed the study?

This study has received a favourable opinion from the West Midlands Black Country Research Ethics Committee.

Further information and contact details

If you require further information about taking part in a clinical study, the following link can assist you. It provides information about how clinical trials are run, and what to expect if you take part in a trial:

http://www.nhs.uk/conditions/Clinical-trials/Pages/Introduction.aspx

If you would like to know more about this study, or if you have any questions, you can contact the study team.

Study team contact details:

- **Email**: define@lindushealth.com
- **Phone:** 020 8609 4690

If you would like advice about whether you should participate in the study, you may want to contact your GP surgery or the study team. It is often useful to discuss the study with your friends and family. Thank you for reading this information sheet, and for considering taking part in the study.
