# Supplementary material for: Digital thErapy For Improved tiNnitus carE Study (DEFINE): Protocol for a randomised controlled trial
Source: PLoS One. 2024 Jan 5;19(1):e0292562. doi: 10.1371/journal.pone.0292562 (PMC10769067; doi:10.1371/journal.pone.0292562)
Supplement: S4 File — ‘Informed consent form for the trial’. (PDF) [file pone.0292562.s005.pdf]

IRAS ID: 328487

Participant Identification Number for this trial:

CONSENT FORM

Title of Project: Digital thErapy For Improved tiNnitus carE (DEFINE)

		Please initial box
1	I confirm that I have read the information sheet dated,28/09/2023, for the above study. I have had the opportunity to consider the information, ask questions and have had these answered satisfactorily.	
2	I understand that relevant sections of my medical notes and data collected during the study, may be looked at by individuals from Lindus Health, Oto Health Ltd or regulatory authorities, where it is relevant to my taking part in this research. I give permission for these individuals to have access to my records.	
3	I consent to being contacted by the research team for the purposes of trial follow up and I understand that this will require me to provide my contact details to the research team and organisations supporting the research team to deliver the trial.	
4	I agree to my General Practitioner being informed of my participation in the study. / I agree to my General Practitioner being involved in the study, including any necessary exchange of information about me between my GP and the research team.	
5	I consent to Lindus Health Limited using my data to improve, develop and create new products and services. This may include using this data to:<br>● improve the study that I am taking part in;<br>● improve planning for future studies;<br>● create statistical analysis, build digital control groups and AI models.	
6	I understand that my personal data will be retained for at least 12 months from trial completion before it is deleted, or longer if required by regulation related to clinical trial activity. For example, any research documents with personal information, such as consent forms, will be held securely for 10 years after the end of the study as per trial regulation.	
7	I understand that my participation is voluntary and that I am free to withdraw at any time without giving any reason, without my medical care or legal rights being affected. I agree to take part in the above study and if I choose to withdraw, data already collected will continue to be used.	
8	I agree to take part in the above ethically approved trial.	

9	I understand that the information collected about me will be used to support other ethically approved research in the future, and may be shared anonymously with other researchers.	
	**ADDITIONAL (optional, not required for study participation)**	
10	I am happy to be contacted by the research team to be invited to Focus Groups / Semi-Structured Interviews at 3, 6 and 12 months.<br><br>(Taking part in the Focus Group or Semi-Structured Interview is optional and will not affect your study participation. If you agree to be contacted, the research team may contact you with details. You can then decide whether you want to take part or not)	

Name of Participant | Date | Signature

Name of Person seeking consent | Date | Signature

1 copy of the electronic informed consent form will be sent to the participant

and

1 copy of the electronic informed consent form will be stored in the site file
