# Supplementary material for: Digital thErapy For Improved tiNnitus carE Study (DEFINE): Protocol for a randomised controlled trial
Source: PLoS One. 2024 Jan 5;19(1):e0292562. doi: 10.1371/journal.pone.0292562 (PMC10769067; doi:10.1371/journal.pone.0292562)
Supplement: S5 File — ‘Topic guide for the qualitative section of the DEFINE trial’. (DOCX) [file pone.0292562.s006.docx]

**Digital thErapy For Improved tiNnitus carE Study  (DEFINE)**

**Topic Guide**

**Design:**

The focus group will be comprised of adults who have participated in the DEFINE trial and will be semi-structured based on the topic guide below. A facilitator will explore the experience and view of the participants, and the transcript will be transcribed, with a thematic analysis employed to understand key topics.

**Broad themes:**

1. Barriers to seeking and obtaining advice and treatment for tinnitus
2. Participant ideas, concerns and expectations about the trial interventions
3. Participants interaction with the trial interventions
4. Suggested improvements to the delivery or content of the trial interventions
5. How the trial interventions would best be deployed within the NHS

| **Main questions** | **Optional probe questions** |
| --- | --- |
| What enabled or prompted you to obtain treatment for your tinnitus? | What happened when you first had tinnitus?  Did you have any knowledge about tinnitus before it happened?  Did you encounter anything or anyone that prompted you to seek help for your tinnitus?  Tell me about any healthcare professionals who helped you access information and treatment?  What was your experience with your GP/primary care? |
| What was your experience of the trial treatment? | What were you expecting in terms of tinnitus treatment?  What is your opinion of the treatment you had?  Was there anything about the treatment that you particularly liked or didn’t like?  Was there anything about the treatment that worried or concerned you?  Did the treatment meet your expectations?  Did you find the treatment easy to complete?  Was it easy to find time or a place to complete the treatment?  Did you find it easy to arrange a hearing test with a high street audiologist and how was this service?  Do you feel you got the support you needed?  Would you recommend the treatment you had to someone else with tinnitus? |
| What could be improved? | Could any aspect of the treatment be improved?  Specifically, what would make the app/F2F treatment easier to use, or better meet your needs?  Did you find the trial easy to understand and complete assessments for? |
| How do you think tinnitus treatment would be best provided within the NHS for people like you? | Do you think tinnitus care is best delivered remotely one to one or via an application?  How important is access to immediate treatment for tinnitus?  Do you think the tinnitus therapy you received is a good way to deliver treatment in the NHS? |
